# Cooperation across healthcare service levels for medication reviews in older people with polypharmacy admitted to a municipal in-patient acute care unit (The COOP II Study): study protocol for a randomized controlled trial

**DOI:** 10.1186/s13063-024-08442-w

**Published:** 2024-09-13

**Authors:** Leonor Roa Santervas, Torgeir Bruun Wyller, Eva Skovlund, Janicke Liaaen Jensen, Katrine Gahre Fjeld, Lene Hystad Hove, Ingrid Beate Ringstad, Lena Bugge Nordberg, Kristin Mæland Mellingen, Espen Saxhaug Kristoffersen, Rita Romskaug

**Affiliations:** 1 Department of Geriatric Medicine, Oslo University Hospital, Oslo, Norway; 2 City of Oslo Health Agency, Municipality of Oslo, Oslo, Norway; 3 Department of Geriatric Medicine, Institute of Clinical Medicine, University of Oslo, Oslo, Norway; 4 Department of Public Health and Nursing, The Norwegian University of Science and Technology, Trondheim, Norway; 5 Department of Oral Surgery and Oral Medicine, Institute of Clinical Dentistry, University of Oslo, Oslo, Norway; 6 Department of Cariology and Gerodontology, Institute of Clinical Dentistry, University of Oslo, Oslo, Norway; 7 REMEDY Centre for treatment of Rheumatic and Musculoskeletal Diseases, Diakonhjemmet Hospital, Oslo, Norway; 8 Department of General Practice, Institute of Health and Society, University of Oslo, Oslo, Norway; 9 Department of Neurology, Akerhus University Hospital, Lørenskog, Norway

**Keywords:** Polypharmacy, Inappropriate drug use, Medication review, Primary health care, General practitioner, Geriatrics, Elderly, Health-related quality of life, Oral health, Carbon footprint

## Abstract

**Background:**

Polypharmacy and inappropriate drug use are associated with adverse health outcomes in older people. Collaborative interventions between geriatricians and general practitioners have demonstrated effectiveness in improving clinical outcomes for complex medication regimens in home-dwelling patients. Since 2012, Norwegian municipalities have established municipal in-patient acute care (MipAC) units, designed to contribute towards reducing the number of hospital admissions. These units predominantly serve older people who typically benefit from multidisciplinary approaches. The primary objective of this study is to evaluate the effect of cooperative medication reviews conducted by MipAC physicians, supervised by geriatricians, and in collaboration with general practitioners, on health-related quality of life and clinical outcomes in MipAC patients ≥ 70 years with polypharmacy. Additionally, the study aims to assess the carbon footprint of the intervention.

**Methods:**

This is a randomized, single-blind, controlled superiority trial with 16 weeks follow-up. Participants will be randomly assigned to either the control group, receiving usual care at the MipAC unit, or to the intervention group which in addition receive clinical medication reviews that go beyond what is considered usual care. The medication reviews will evaluate medication appropriateness using a structured but individualized framework, and the physicians will receive supervision from geriatricians. Following the clinical medication reviews, the MipAC physicians will arrange telephone meetings with the participants’ general practitioners to combine their assessments in a joint medication review. The primary outcome is health-related quality of life as measured by the 15D instrument. Secondary outcomes include physical and cognitive functioning, oral health, falls, admissions to healthcare facilities, and mortality.

**Discussion:**

This study aims to identify potential clinical benefits of collaborative, clinical medication reviews within community-level MipAC units for older patients with polypharmacy. The results may offer valuable insights into optimizing patient care in comparable municipal healthcare settings.

**Trial registration:**

The study was registered prospectively on ClinicalTrials.gov 30.08.2023 with identifier NCT06020391.

## Administrative information

Note: the numbers in curly brackets in this protocol refer to SPIRIT checklist item numbers. The order of the items has been modified to group similar items (see http://www.equator-network.org/reporting-guidelines/spirit-2013-statement-defining-standard-protocol-items-for-clinical-trials/).

**Table:** 

Title {1}	Cooperation across healthcare service levels for medication reviews in older people with polypharmacy admitted to a municipal in-patient acute care unit (The COOP II Study): study protocol for a randomized controlled trial.
Trial registration {2a and 2b}.	ClinicalTrials.gov. Identifier: NCT06020391.
Protocol version {3}	Version 1.5. February 5, 2024.
Funding {4}	The study is funded by the South-Eastern Norway Health Authorities (grant number 2022066).
Author details {5a}	Leonor Roa Santervas^1,2,3^, Torgeir Bruun Wyller^1,3^, Eva Skovlund^4^, Janicke Liaaen Jensen^5^, Katrine Gahre Fjeld^6^, Lene Hystad Hove^6^, Ingrid Beate Ringstad^5^, Lena Bugge Nordberg^7^, Kristin Mæland Mellingen^2^, Espen Saxhaug Kristoffersen^8,9^, Rita Romskaug^1^. ^1^ Department of Geriatric Medicine, Oslo University Hospital, Oslo, Norway ^2^ City of Oslo Health Agency, Municipality of Oslo, Oslo, Norway ^3^ Department of Geriatric Medicine, Institute of Clinical Medicine, University of Oslo, Oslo, Norway ^4^ Department of Public Health and Nursing, The Norwegian University of Science and Technology, Trondheim, Norway ^5^ Department of Oral Surgery and Oral Medicine, Institute of Clinical Dentistry, University of Oslo, Oslo, Norway ^6^ Department of Cariology and Gerodontology, Institute of Clinical Dentistry, University of Oslo, Oslo, Norway ^7^ REMEDY Centre for treatment of Rheumatic and Musculoskeletal Diseases, Diakonhjemmet Hospital, Oslo, Norway ^8^ Department of General Practice, Institute of Health and Society, University of Oslo, Oslo, Norway ^9^ Department of Neurology, Akerhus University Hospital, Lørenskog, Norway.
Name and contact information for the trial sponsor {5b}	Rita Romskaug (Principal Investigator). Department of Geriatric Medicine, Oslo University Hospital. E-mail: ritrom@ous-hf.no
Role of sponsor {5c}	This is an investigator-initiated clinical trial. The sponsor, Oslo University Hospital, takes responsibility for the design of the study, the collection, analysis, and interpretation of the data, and the writing of the manuscript. The design, management, analysis and reporting of the study are entirely independent of the funding source.

## Introduction

### Background and rationale {6a}

Today’s elderly population often relies on multiple medications [1, 2]. While this can be necessary and beneficial, it also increases the risk of inappropriate treatment and adverse drug effects [3]. In Norway, general practitioners (GPs) are the main contact for patients and prescribe most medications. They are trained to provide high-quality care for people of all ages, but often face challenges when treating older patients with multimorbidity [4, 5]. Most therapeutic guidelines used in primary care are created for patients with a limited number of comorbid conditions, potentially making them less relevant for frail individuals with multiple illnesses [6, 7]. Geriatricians are trained to address complex medical needs in older patients, but their services are limited, and the shift towards shorter hospital stays and fewer hospital beds contribute to an increasing amount of medical care for complex patients being transferred from hospitals to local municipalities.

The Coordination Reform in Norway [8] was implemented from 2012, and a central aim was to achieve better coordination for patients who are frequently transferred between different levels of care. As part of this reform, all Norwegian municipalities are imposed to establish municipal in-patient acute care (MipAC) units. These primary health care services are designed to contribute towards reducing the number of acute hospital admissions [9]. There is a need for scientifically rigorous studies to determine how best to utilize the MipAC units. Additionally, research on effective collaboration and supervision methods between different levels of health care is vital.

Although numerous intervention studies aiming at optimizing medication use in older people have been conducted, the majority emphasize surrogate endpoints with limited clinical relevance [10]. The COOP study (Cooperation for Improved Pharmacotherapy in Older People with Polypharmacy) demonstrated that a collaboration between geriatricians and GPs on clinical medication reviews can significantly improve older patients’ health-related quality of life (HRQoL), along with their physical and cognitive function [11, 12]. However, only a scarcity of research directly measures the impact of such interventions on clinical outcomes. Further, oral health issues tend to escalate with age, often significantly impacting on nutrition and quality of life [13, 14], and even increasing mortality [15]. The need to see oral and general health in context has recently been pointed out by The World Health Organization [16] as well as by leading international researchers [17]. The interplay between polypharmacy, medication optimization, and oral health remains inadequately explored, with a marked deficit in research addressing the prevention and management of oral side effects associated with polypharmacy [18]. Building on this research gap, the COOP II study will also incorporate oral health indicators as outcomes of the intervention.

Climate change, driven by human activity, is the greatest health threat facing humanity according to the World Health Organization [19], and the health care sector is a major contributor to global greenhouse gas emissions [20, 21]. Clinical care pathways ought to balance effectiveness and patient safety with a commitment to environmental sustainability. Reflecting this imperative, this study will extend its analytical scope to assess the environmental impact of the intervention.

### Objectives {7}

The study’s main objective is to assess the impact of cooperative medication reviews conducted by MipAC physicians, under the supervision of geriatricians and in collaboration with GPs, in MipAC patients aged ≥ 70 years with polypharmacy. The primary outcome is HRQoL measured by 15D instrument. Secondary outcomes include physical and cognitive function, oral health, admissions to healthcare facilities, and mortality. The study will also evaluate the intervention’s carbon footprint compared to usual care.

### Trial design {8}

**Fig. 1:**
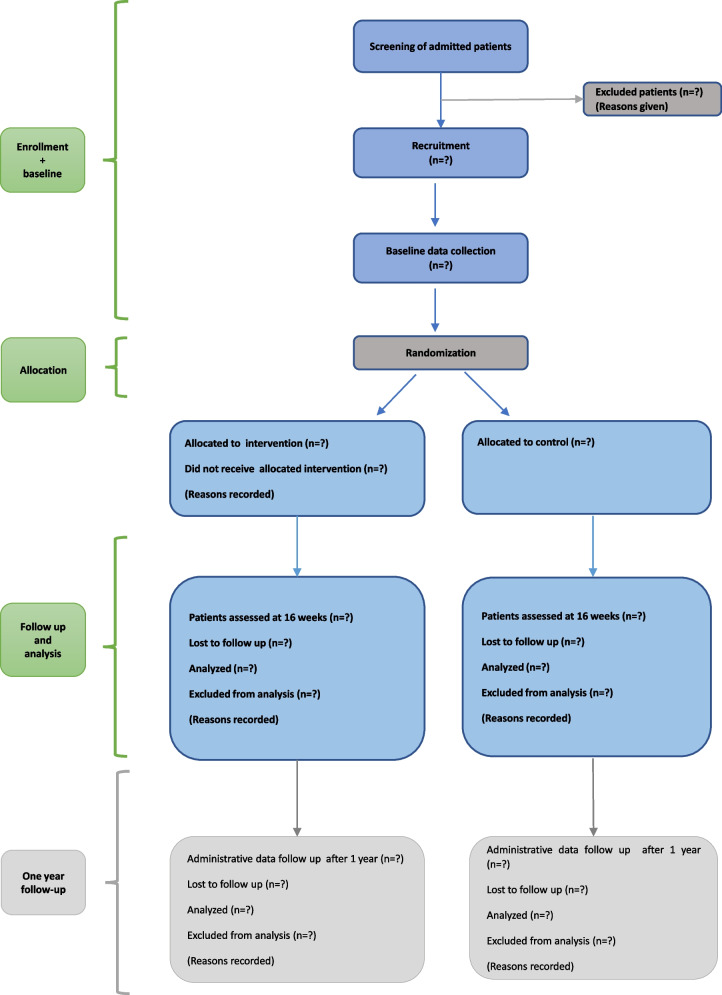
Flowchart of the study’s design

This is a randomized, single-blind, controlled, superiority trial. The individual participant constitutes the randomization unit, with allocation distributed in a 1:1 ratio with two parallel groups. The follow-up period is 16 weeks (±2 weeks) for the clinical outcomes. Moreover, we aim to collect data on mortality and admissions to healthcare facilities 1 year following enrolment. A flowchart of the study’s design is shown in Fig. 1.

## Methods: participants, interventions and outcomes

### Study setting {9}

The study will be conducted at the MipAC unit in Oslo, Norway. This unit, which has 72 beds, admits approximately 4000 patients per year.

### Eligibility criteria {10}

Patients admitted to the MipAC unit, aged ≥ 70 years, using at least six different systemic medications regularly (including preparations for inhalation, vitamin supplements, and laxatives but excluding topical drugs like eye drops and ointments), and who are capable of providing informed consent, are considered eligible for the study.

Exclusion criteria are as follows: previously included in the study, not speaking or understanding Norwegian, residing outside of the municipality of Oslo, planned discharge within 24 h, isolated for infection control reasons, life expectancy judged to be less than 6 months, considered too ill to participate, or presence of specific concerns by MipAC personnel that are not addressed by the other exclusion criteria.

### Who will take informed consent? {26a}

MipAC personnel will screen potentially eligible patients for inclusion/exclusion criteria. Patients fulfilling the criteria and accepting to receive information about the study will be informed about the study by a research assistant (RA) and asked for informed consent.

The MipAC population represents a vulnerable group where some will have dementia or cognitive impairment. As a result, the ability of these patients to provide informed consent may be limited, and we will place special emphasis on providing comprehensive information and assessing their competence to give informed consent.

### Additional consent provisions for collection and use of participant data and biological specimens {26b}

Participants provide informed consent also for the planned data collection 1 year following enrolment.

## Interventions

### Explanation for the choice of comparators {6b}

The control group will receive “usual care” at the MipAC unit. This encompasses a broad spectrum of assessments and medical interventions, each tailored to the individual patient’s specific needs. While attending physicians may seek to optimize the general medication regimen also for some patients in the control group, the nature of any medication review in this arm will vary, reflecting the diverse clinical practices among different physicians at the MipAC unit. Such reviews will not include the structured approach characteristic of the intervention arm, which involves systematic discussions with geriatricians and GPs to improve medication management. This choice of comparator aligns with the study’s primary objective of evaluating the effect of the intervention. Usual care at the MipAC unit offers the most direct comparison and is ethically acceptable.

### Intervention description {11a}

The intervention consists of three main parts: (1) clinical assessment of the participant and medication review by a MipAC intervention physician, (2) supervision by geriatrician, and (3) a telephone meeting between the MipAC physician and the participant’s GP.

1. Clinical assessment and medication review

We aim to recruit 1–4 MipAC intervention physicians who are specialized in General Practice to take part in the study, and patients randomized to the intervention group will be assigned to one of them as their attending physician. In addition to providing usual care, these intervention physicians will conduct structured clinical medication reviews aimed at optimizing the patients’ total medication use.

The framework for conducting the medication reviews draws upon previous experiences from the previous COOP study, incorporating recommendations from the Norwegian Directorate of Health’s guidelines on medication reviews and polypharmacy [22], as well as the Scottish NHS’s polypharmacy guidance [23]. The cornerstone of the medication reviews is a personalized approach, tailored to each patient’s unique combination of health conditions, symptomatology, medication use, and individual preferences.

The MipAC intervention physician will obtain information regarding the patient’s medical history and medication use, ensure the availability of necessary supplementary diagnostics (e.g., blood analyses, electrocardiograms, and blood pressure measurements), and conduct a physical examination. All medications in use will be approached systematically to ensure medication appropriateness, optimize disease control, ensure correct dosing, and reduce the risk of adverse effects and drug interactions. Tools such as drug interaction databases will be employed routinely to support this process [24]. Furthermore, explicit tools like lists of anticholinergic drugs [25, 26], STOPPFrail [27, 28], and STOPPFall [29] may be consulted; however, these tools are supplementary rather than integral to the medication review process.

(2) Geriatric supervision

For clinical supervision, the MipAC intervention physician will consult a geriatrician (RR; TBW) to review patient findings and proposed medication adjustments. They will discuss the optimal timeline for these adjustments and evaluate any needs for new medications. This consultation will take place prior to the meeting with the patient’s GP.

(3) Telephone meeting between the MipAC intervention physician and the FP

After conducting the clinical medication review, the MipAC intervention physician will arrange a telephone meeting with the patient’s GP to discuss the findings. This collaborative medication review aims to optimize the patient’s medication plan, considering both the geriatric insights and the GP’s knowledge of the patient’s history. The two physicians will discuss potential adjustments as well as the patient’s need for further follow-up, and collaboratively develop a step-by-step plan for adjusting medications. This interaction can alternatively be conducted via digital communication platforms according to the GP’s preferences. If for any reasons direct contact is not established, the GP will receive a written summary of the clinical medication review (steps 1 and 2).

### Criteria for discontinuing or modifying allocated interventions {11b}

Allocated interventions will neither be discontinued nor modified. Usual care does not inherently entail risks and may sometimes include components of the intervention. The intervention will be administered by designated MipAC intervention physicians who are also familiar with routines at the MipAC unit, ensuring that usual care is consistently provided. Inability to establish contact with the GP will not be considered a reason for modifying or discontinuing the allocated interventions since the medication review will still be conducted in collaboration with the geriatrician, and the GP will be informed through written documentation.

### Strategies to improve adherence to interventions {11c}

Patients in the intervention group receive a structured discharge conversation focused on medication management. Any modifications to their medication regimen are recorded in the medication system, home care services (if relevant) are informed, and a review summary is sent to their GP. For participants with multiple medication adjustments or other clinical indications, a follow-up appointment with their GP will be recommended.

### Relevant concomitant care permitted or prohibited during the trial {11d}

Participants will resume their usual care after discharge. There will be no recommendations regarding concomitant care. The GP remains responsible for post-discharge care decisions and will assess participant needs independently of the study.

### Provisions for post-trial care {30}

Compensation for individuals who may suffer harm related to the MipAC unit admission will be covered by the Norwegian Patient Injury Act independently of participation in the study.

### Outcomes {12}

#### Primary outcome

The primary outcome measure is HRQoL, measured by the 15D instrument at 16 weeks, adjusted for baseline score [30]. The 15D instrument is a validated patient-reported outcome measure that has been used in similar geriatric interventions [31, 32], including the previous COOP study [12]. It encompasses 15 dimensions including mobility, vision, hearing, breathing, sleeping, eating, speech, elimination, usual activities, mental function, discomfort and symptoms, depression, distress, vitality, and sexual activity [33]. Each dimension is rated on a five-level ordinal scale, with the respondents choosing the level that best describes their present health status. The 15D instrument offers both a profile measure and a single index representing overall HRQoL. For the purpose of this study, the single index version will be utilized. Scores are calculated by population-based utility weights and range from 0 (poorest HRQoL) to 1 (excellent HRQoL) [30, 33]. A change of ± 0.015 or more is considered the minimum important change (MIC), and a change of more than 0.035 in the positive direction represents “much better HRQoL” [34]. Since acute illness can impact HRQoL, patients are asked at baseline to provide responses reflecting their typical condition prior to the acute episode that led to admission.

#### Secondary outcomes

Secondary outcomes are assessed 16 weeks after baseline and include physical and cognitive function, oral health, falls, admissions to healthcare facilities, and mortality. Details on these outcomes are listed in Table 1.

**Table 1:** Detailed description of secondary outcomes

**Outcome**	**Description**
Handgrip strength [35]	Assessed using a standard dynamometer, with three attempts for each hand. The highest value from all six attempts will be reported, measured in kilograms.^a^
Orthostatic blood pressure	Supine blood pressure and pulse rate will be measured after a minimum of 5 min of rest. The patient will then stand up, and measurements will be repeated after 1 and 3 min. Orthostatic hypotension will be defined as a fall in systolic blood pressure of at least 20 mmHg or a fall in diastolic blood pressure of at least 10 mmHg. We will report both the percentage of patients exhibiting orthostatic hypotension and blood pressure values in mmHg.^a^
Digit span forward and backward [36]	Results will be presented as the maximum digit span achieved, reported separately for digit span forward and backward.^a^
Shortened Xerostomia Inventory [37]	A five-item summated rating scale which combines the responses to five individual items into a single sum score. The sum score can range from 5 to 15, with higher values representing more severe xerostomia.^b^
Unstimulated whole saliva secretion rate [38]	Unstimulated whole saliva will be measured over a 3-min period. Prior to the initiation of the test, patients will be instructed to swallow their saliva. During the collection phase, they will be asked to allow any saliva produced to passively drip into a plastic cup. The plastic cup will be weighed both before and after the collection period. Results will be reported in milliliters per minute.^a^
The eight-item visual analog scale xerostomia questionnaire [39]	Visual analog scale with eight questions related to xerostomia. Participants will be asked to mark their response to each item by placing a vertical line on the 100-mm horizontal scale [38]. Results will be reported in millimeters.^a^
Oral pain/discomfort	The patients will be asked if they have experienced any oral pain/discomfort over the last 3 months (baseline) and since discharge from the index MipAc unit stay until week 16. Responses will be given on a five-category rating scale ranging from “never” (0), “hardly ever” (1), “occasionally” (2), “often” (3), to “very often” (4).^b^
Falls	Number of falls since discharge from the index MipAC unit stay until week 16.
Hospital/MipAC unit admissions	The total count of hospital admissions and readmissions to the MipAC unit since discharge from the index MipAC unit stay until week 16.
Mortality	Mortality in the period from enrolment until week 16.

*Abbreviations: MipAC *Municipal in-patient Acute Care

^a^The measurement may be affected by acute conditions present during the MipAC unit stay. Comparison between the intervention and control group will be made at week 16, without adjusting for baseline values

^b^The measurement is anticipated to be minimally affected by acute conditions present during the MipAC unit stay. Comparison between the intervention and control group will be made at week 16, adjusting for baseline values

#### Other pre-specified outcomes

We will calculate the carbon footprint of the intervention and usual care by conducting a life cycle assessment based on ISO standards [40]. We will include data such as types and quantities of medications administered, types and quantities of medical equipment used during the MipAC unit stay, number and length of admissions to healthcare facilities, and number of outpatient and GP consultations in the period from discharge until 16 weeks. These data will be converted to carbon dioxide equivalents.

The primary study period will end after 16 weeks. However, we will also evaluate long-term outcomes in terms of mortality rates and admissions to healthcare facilities up to 1 year.

#### Descriptive variables

- Demographics and diagnoses according to ICD-10.
- Charlson Comorbidity Index (CCI) [41].
- Clinical Frailty Scale (CFS) [42].
- Oral health: Number of teeth, tooth mobility, number of patient-referred implants, the Revised Oral Assessment Guide [43], and the Mucosal-Plaque Score [44].
- Medication details: Information on medications utilized at baseline and after 16 weeks, medication discrepancies, changes to medications initiated during admittance and post-discharge, and recommendations on medication adjustments outlined in the discharge letter. Medications will be registered according to the Anatomical Therapeutic Chemical classification system [45].
- Nutritional status: Measurement of mid-upper arm circumference [46].
- Details on the MipAC unit stay: Number of attending physicians, length of stay, reasons for admittance, discharge diagnoses, severity of acute illness as assessed by National Early Warning Score 2 (NEWS2) scores [47, 48], and types and quantities of medical equipment used.
- Post-discharge healthcare service utilization: This will encompass the frequency of consultations with FPs, the number of outpatient visits, and admissions to various healthcare facilities including hospitals, MipAC units, nursing homes, and rehabilitation institutions.

### Participant timeline {13}

**Fig. 2:**
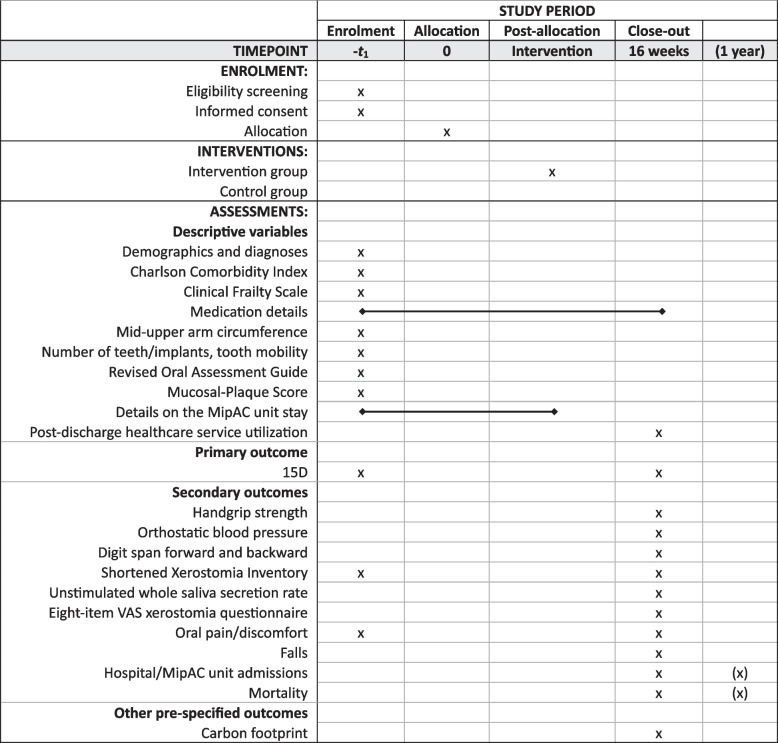
Schedule of enrolment, interventions and assessments

The participant timeline is illustrated in Fig. 2.

### Sample size {14}

Based on the results of the previous COOP study, we hypothesize a mean difference between groups of 0.04 on 15D, and the standard deviation of the change in 15D from baseline to 16 weeks is assumed to be 0.13 [12]. With inclusion of a total of 350 patients (whereof 175 in the intervention group), the power to detect a difference of 0.04 on 15D will be at least 80% with a significance level of 5% [11].

### Recruitment {15}

The recruitment team combines the clinical expertise of health personnel familiar with the MipAC setting with the research experience of those skilled in clinical trial recruitment. This ensures efficient and effective participant enrolment.

## Assignment of interventions: allocation

### Sequence generation {16a}

The allocation sequence is computer-generated using variable block randomization. Details on block sizes are concealed from study personnel. No stratification factors are used.

### Concealment mechanism {16b}

Participant registration, data collection, and randomization are seamlessly integrated within the Viedoc™ system. The randomization procedure, along with the outcomes of the randomization, is concealed from the blinded RAs within Viedoc™ due to restrictions defined in their user profiles.

### Implementation {16c}

A statistician independent of the study team (Clinical Trial Unit, Oslo University Hospital) prepared the allocation sequence. RAs enrol participants. LRS performs the randomization within Viedoc™ after completion of baseline assessments.

## Assignment of interventions: blinding

### Who will be blinded {17a}

The RA conducting all assessments is blinded to participant allocation. Due to the intervention’s nature, blinding of participants, relatives, or GPs is not feasible. A blinded statistician will analyze the primary outcome.

### Procedure for unblinding if needed {17b}

We do not foresee any circumstances in which the RAs or statistician need to be unblinded.

## Data collection and management

### Plans for assessment and collection of outcomes {18a}

The participants will be assessed during their MipAC unit admission and at the home visit 16 weeks after baseline examinations. We will collect data on diagnoses, comorbidities, medications, and healthcare utilization from various sources: the participant, MipAC unit records, the Norwegian centralized patient journal, hospital records, and home nursing care service records. All personnel collecting data will be properly trained, and the investigators will review data for accuracy.

### Plans to promote participant retention and complete follow-up {18b}

Upon enrolment, participants are provided with both oral and written information about follow-up procedures. Thirteen weeks after baseline examinations, the RA will contact the participants to schedule the 16-week visit. Participants will also receive a reminder call 24 h prior to assessment. Should participants reject to conduct the home visit, they are given the option to submit their 15D scores and medication information by phone. Collected data from participants who withdraw their consent will be deleted if they request so.

### Data management {19}

Non-identifiable source data are paper versions of the Case Report Forms (CRFs). Data will be entered into electronic Case Report Forms (eCRFs) within the Viedoc™ system direct after patient assessments. These eCRFs, designed by the Clinical Trial Unit at Oslo University Hospital, incorporate automatic range checks and missing data alerts to enhance data quality. Information regarding admissions to healthcare facilities, NEWS2 scores and mortality are directly plotted into spreadsheets.

### Confidentiality {27}

Data will be stored on secure, access-restricted research servers at Oslo University Hospital and the University of Oslo. Each participant will receive a unique identification number. The code linking participants to their identification number will be securely maintained within Medinsight, a system for creating customized registers, on access-restricted servers at Oslo University Hospital. Paper-based materials will be securely stored in locked file cabinets within restricted-access facilities, accessible only to authorized researchers within the research group. Only anonymized data will be published. Person-linkable data will be stored until the end of the project on September 5, 2033, and thereafter deleted.

### Plans for collection, laboratory evaluation and storage of biological specimens for genetic or molecular analysis in this trial/future use {33}

N/a. No biological specimens will be collected for future use.

## Statistical methods

### Statistical methods for primary and secondary outcomes {20a}

#### Primary outcome

The primary analysis will utilize an intention-to-treat approach where all subjects will be maintained in the treatment group to which they were initially assigned.

The primary outcome measure 15D is on an interval scale and is expected to be reasonably normally distributed [30, 33]. This measure will be analyzed using Analysis of Covariance (ANCOVA), with 15D score at week 16 as the dependent variable, randomization group as the fixed factor, and baseline 15D score as covariate. A *p* value of less than 0.05 will be considered indicative of the difference between groups being statistically significant. Differences between treatment groups will be estimated using 95% confidence intervals.

#### Secondary outcomes

To compare the intervention and control group with respect to the secondary outcomes, we will employ various statistical methods based on the characteristics of each variable. These methods include ANCOVA for repeated measures of continuous data, logistic regression for binary outcomes, and independent samples *t*-tests for continuous variables that are measured only after 16 weeks. For non-normally distributed variables, we will apply suitable transformations, such as logarithmic or square root adjustments, to improve normality before analyses. Alternatively, these variables may be analyzed using non-parametric tests if transformations do not result in normal distribution. Regarding the secondary outcomes, we will not implement imputation techniques for missing data, nor will we adjust *p* values for multiple testing.

### Interim analyses {21b}

N/a. No interim analysis is planned.

### Methods for additional analyses (e.g., subgroup analyses) {20b}

#### Adjusted analysis of the primary outcome

Variables known or presumed to have a prognostic impact on the outcome will be individually included as covariates in the analysis, alongside baseline 15D score and randomization group. Should the inclusion of these variables alter the effect estimate for the randomization factor with 10% or more, they will be incorporated into a final model that includes all variables demonstrating an effect of this magnitude. The following variables will be subject to such analyses: age, sex, CFS, CCI, and living alone (yes/no).

#### Responder analyses

In addition to comparing mean differences in 15D scores between groups, we plan to conduct responder analyses to examine the intervention’s impact from various perspectives. This approach involves categorizing patients as “responders” if they exhibit an improvement of at least 0.015 (MIC) on the 15D scale. By doing so, we will identify the proportion of individuals experiencing meaningful enhancements in their HRQoL as a result of the intervention.

### Methods in analysis to handle protocol non-adherence and any statistical methods to handle missing data {20c}

#### Missing data on 15D

To calculate the 15D score, responses are required for every question (dimension). In instances where up to three responses are missing, we will apply the imputation algorithm provided by the developers of the instrument [49]. Should an observation contain more than three missing values, the 15D score will be classified as missing and addressed through multiple imputation using the mi procedure in Stata with M=20 imputations.

#### Lost to follow-up

Participants who die before follow-up will be assigned a score of “0” (representing the worst possible HRQoL) on the 15D scale. Participants who are lost to follow-up for other reasons than death will be included in the primary analysis, and missing values addressed through multiple imputation as described above.

#### Sensitivity analyses

Missing values for the primary outcome will be examined through various analytical methods to assess their potential impact on the study results:

- Analysis 1: Participants not handled according to randomization and participants that are missing (all reasons) will be excluded (per protocol analysis).
- Analysis 2: Participants missing for other reasons than death will be excluded, but deceased participants will be kept with the value “0” on 15D.
- Analysis 3: Participants missing for other reasons than death will be handled as “last observation carried forward”, but deceased participants will be kept with the value “0” on 15D.

### Plans to give access to the full protocol, participant-level data and statistical code {31c}

Data and statistical code will be made available upon reasonable request to the principal investigator (RR), in accordance with regulations set forth by The Regional Committee for Medical & Health Research Ethics in Norway.

## Oversight and monitoring

### Composition of the coordinating center and trial steering committee {5d}

The Department of Geriatric Medicine at Oslo University Hospital represents the coordinating center of the study. LRS and two RAs are responsible for the day-to-day operation of the study. LRS assists the research team in making daily decisions, manages participant allocation, performs interventions, supports other MipAC physicians in carrying out interventions, ensures accuracy of baseline data, prepares information for follow-up visits, and collects data on adverse events. The RAs enrol participants, collect baseline data, coordinate 16-week follow-ups, and conduct the follow-up visits. The principal investigator (RR) maintains ongoing communication with LRS, overseeing the study’s overall coordination and provides both scientific and administrative guidance. In addition, a senior researcher (TBW) provides close supervision to the research team. RR, LRS, and TBW communicate at least weekly to monitor trial progress.

### Composition of the data monitoring committee, its role and reporting structure {21a}

No data monitoring committee will be established for this study. The responsibility for overseeing data resides with the principal investigator. This process is completely independent of the funding source.

### Adverse event reporting and harms {22}

We consider the risk to participants in the study to be minimal, as the intervention involves a presumably more thorough medication review than what would otherwise be conducted. However, we cannot completely exclude the possibility of participants experiencing adverse drug reactions if new medications are introduced, or adverse withdrawal events if medications are discontinued. We will register all adverse effects that may be associated with the intervention. Should study personnel identify potentially serious problems with a participant’s medication regimen during data collection, this information will be communicated to their attending MipAC physician or GP, regardless of allocation group.

### Frequency and plans for auditing trial conduct {23}

The research team will maintain continuous communication throughout the study, discussing participant including strategies, ethical challenges, and methodological issues. We do not anticipate the need for a separate auditing plan for trial conduct.

### Plans for communicating important protocol amendments to relevant parties (e.g., trial participants, ethical committees) {25}

Significant protocol amendments will only be implemented following approval from the ethical committee.

### Dissemination plans {31a}

The study’s results are intended for peer-reviewed scientific journals, with an expectation of producing publications in high-impact journals within the fields of general medicine, geriatrics, general practice, dentistry, or health services research. In addition, we expect to present results as posters or oral presentations at relevant conferences. Pertinent data related to quality improvement will be communicated to healthcare professionals at the MipAC unit as well as to Norwegian healthcare authorities to enhance patient safety.

## Discussion

Older patients face a higher prevalence and risk of polypharmacy compared to other age groups [1]. While many medications are essential for managing multiple health conditions, it is crucial to ascertain which medications are truly necessary and which may pose harm to individual patients [6].

The previous COOP study [12] demonstrated the effectiveness of collaborative medication reviews conducted by geriatricians in partnership with GPs for elderly home-dwelling patients, with significant enhancements in HRQoL. However, with an aging population and limited healthcare resources, it becomes imperative to prioritize interventions that optimize medication use while considering the constraints of available professional expertise and economic resources. Our decision to assess clinical outcomes subsequent to collaborative medication reviews in elderly patients with polypharmacy admitted to a MipAC unit is designed to give new insights into possible clinical improvements, the importance of cross-level collaboration within the healthcare system, and its potential implications for environmental sustainability.

There are some limitations of the study. Firstly, the participants not only exhibit age-related conditions and multiple chronic illnesses, but are also acutely ill at the time of enrolment. This complexity may hinder the full implementation of the intervention and can also make the intervention less powerful. Furthermore, given their circumstances, patients who agree to participate may be those with a clearer understanding of the potential benefits. In contrast, those heavily reliant on medications might hesitate to participate out of fear of the intervention disrupting their medical treatment.

Secondly, the physicians at the MipAC unit who are not MipAC intervention physicians will remain uninformed about patients included in the control group and will not oversee patients in the intervention group. However, there is a possibility of methodological “spillover” where MipAC physicians adopt medication optimization strategies into their usual care practices for the control group. Such unintended diffusion could attenuate the discernibility of the intervention’s true effect. However, considering the short duration of the intervention, the magnitude of this impact is anticipated to be minimal.

Thirdly, in contrast to the previous COOP study, GPs have not actively volunteered to participate in this study. Instead, they will be automatically contacted whenever one of their patients is assigned to the intervention group. While this approach may result in reduced engagement from GPs, and in some instances may end in failure to establish telephone contact, potentially diminishing the effectiveness of the intervention, it may also yield to opposite effect if the GP has encountered challenges with certain medications in the past, leading to improved patient intervention. This nuanced approach aligns more closely with real-world clinical scenarios.

Fourthly, recruiting elderly patients in acute situations for such studies presents significant challenges and may result in inadequate sample sizes [50, 51].

Due to the diverse range of comorbidities, clinical conditions, and personal preferences of each participant, along with their functional status, achieving full standardization of the intervention is neither feasible nor desirable. Consistent with the approach taken in the previous COOP study, our main strategy will involve providing a detailed description of the interventions conducted, with a particular focus on modifications made to individual participants’ medication regimens.

## Trial status

Recruitment started on September 6, 2023. The study is ongoing, and the anticipated time for completed recruitment will be July 2024.The study is aligned with protocol version 1.5, dated February 5, 2024.

## Data Availability

The pseudoanonymized, original dataset will only be available to researchers directly involved in the study, and will be kept on secure, access-restricted research servers. Anonymized data will, upon publication of the study and in accordance with regulations set forth by The Regional Committee for Medical & Health Research Ethics in Norway, be made available upon reasonable request to the principal investigator.

## Abbreviations

ANCOVA: Analysis of covariance
CFS: Clinical Frailty Scale
CCI: Charlson Comorbidity Index
CRFs: Case Report Forms
eCRFs: Electronic Case Report Forms
COOP: Cooperation for Improved Pharmacotherapy in Older People with Polypharmacy
GP: General Practitioner
HRQoL: Health-related quality of life
MIC: Minimal Important Change
MipAC: Municipal in-patient Acute Care
NEWS: National Early Warning Score
RA: Research assistant
RCT: Randomized controlled trial
RP: Research physician
